# Correlations between gut microbiota and lichen planus: a two-sample Mendelian randomization study

**DOI:** 10.3389/fimmu.2023.1235982

**Published:** 2023-09-12

**Authors:** Ming Yan, Yu-Long Ouyang, Li-Yuan Xiao, Man Ao, Martin Gosau, Reinhard E. Friedrich, Ralf Smeets, Ling-Ling Fu, Hong-chao Feng, Simon Burg

**Affiliations:** ^1^ Department of Oral and Maxillofacial Surgery, Guiyang Hospital of Stomatology, Guiyang, China; ^2^ Department of Oral and Maxillofacial Surgery, University Medical Center Hamburg-Eppendorf, Hamburg, Germany; ^3^ Department of Oral and Maxillofacial Surgery, Division of Regenerative Orofacial Medicine, University Medical Center Hamburg-Eppendorf, Hamburg, Germany

**Keywords:** Mendelian randomization study, gut microbiota, lichen planus, intestine-skin axis, correlations

## Abstract

**Purpose:**

Several existing studies have revealed that the occurrence of lichen planus (LP) is relevant to the gut microbiota, and the causal relationship between gut microbiota and LP was analyzed using the Mendelian randomization (MR) method.

**Methods:**

Through the two-sample MR method, single nucleotide polymorphisms (SNPs) relevant to gut microbiota were selected as instrument variables (IVs) to evaluate the causal association between gut microbiota and the risk of LP.

**Results:**

According to the selection criteria of inverse-variance weighted (IVW), six bacterial genera were found to be significantly linked to the initiation of LP; The IVW results suggested that Oxalobacteraceae, Victivallaceae, and Actinobacteria could restrain the initiation of LP, showing protective effects against LP. Desulfovibrio, Veillonella, and Ruminococcus gauvreauii groups were demonstrated to have casual correlations with the onset of LP.

**Conclusion:**

The relationship between gut microbiota and LP was not a single positive or inverse relationship. Investigation of the causal relationship of these gut microbiota with LP could further provide evidence for the intestine-skin axis theory. However, the specific mechanism of microorganisms affecting the skin remains to be clarified. In this paper, the protective effects and mechanisms of Oxalobacteraceae, Victivallaceae, and Actinobacteria on LP require further exploration.

## 1 Introduction

Lichen planus (LP) is an idiopathic inflammatory skin disorder of unknown etiology, which can involve the skin, mucosa (especially oral mucosa), scalp, fingers, toes, and external genitalia. Among them, oral mucosal diseases are the most common which can ulcerate oral mucosa and Severe cases can cause make it more susceptible to carcinogenesis, especially on the long-term ulcer site. LP is considered a premalignant condition by World Health Organization (WHO) (1), 1%-2% cases malignant transformation to oral squamous cell carcinoma, while the detailed mechanism underlying this process are still obscure (2). When involving the skin, it usually occurs in the inner side of the limbs or throughout the body. The typical skin lesions are manifested with polygonal- or round-shaped purple-red flat papules with clear boundaries and a chronic course (1). Although the etiology and pathogenesis of LP have not been fully elucidated, many scientists believe that LP is mostly related to dysregulation of the immune system (1), and it is a chronic inflammatory autoimmune disease modulated by T cells, with a global incidence of 0.5% - 2.2% (2), most common in middle-aged females.

The gut microbiota is the largest microbial community in the human body. Gut microbiota exerts a significant role in maintaining the balance of the immune and nervous systems in the host, such as the clearance of pathogens, the development of the immune system, and the regulation of the central nervous system (3, 4). Gut microbiota is strongly linked to host immunity, and the activation state of the host and genetic susceptibility can be triggered or motivated by specific microorganisms (3). Disruption of the balance between the host and microbiota can lead to autoimmune diseases such as Sjogren’s syndrome (4), systemic lupus erythematosus (5), and rheumatoid arthritis (6) through different mechanisms.

Microorganisms, which were previously considered pathogenic microorganisms, are generally believed to supply colonization conditions for microorganisms during the initiation and progression of LP but do not result in the initiation and progression of LP. However, dysbiosis of gut microbiota may also play a role in the occurrence of LP (7).

Traditional observational studies are easily affected by reverse causality to produce bias and cannot clarify its causality. Mendelian randomization (MR) is widely applied for etiological inference in the field of genetic epidemiology (8), which can overcome the bias resulting from confounding and reverse causation issues. The exposure files and outcome files in the two-sample MR in this paper are from 2 different cohorts (9). In this study, we intended to reveal the correlation of gut microbiota at the genetic level with the onset of LP through the two-sample MR method based on the genome-wide association study data of gut microbiota and LP, so as to lay the foundation for the formulation of clinical prevention strategies for primary early-stage LP.

## 2 Materials and methods

### 2.1 Data sources

Genetic variations in the gut microbiota were derived from the largest genome-wide analysis of gut microbiota composition published to date by the MiBioGen Consortium (10, 11); their analysis enrolled 18,340 individuals from 24 cohorts, the majority of whom had European ancestry (n=13,266). The microbial composition was described targeting the variable regions V4(n=8472), V3-V4(n=5791), and V1-V2(n=4774) of the 16S rRNA gene, and classified using the direct classifying method. Microbiota quantitative trait loci (mbQTL) mapping analysis was performed to identify host genetic variations mapped by gene loci relevant to the abundance level of bacterial taxa in the gut microbiota. In the current study, the genus was the lowest taxonomic level. There are 131 genera with an average abundance higher than 1%, from which 12 unknown genera were excluded. Finally, 119 bacterial genera were identified. The GWAS summary statistics of LP were sourced from FinnGen (8th data release) (12). The GWAS of “lichen planus” adopted in this study contained 334116 individuals, including 3141 cases and 330975 controls.

### 2.2 Instrumental variables

IVs in MR study must meet three core assumptions: (1) correlation hypothesis: IVs are closely correlated with exposure, with F value > 10 as the standard of a close correlation; (2) Exclusive hypothesis: IVs do not affect outcomes; (3) Independence assumption: IVs are irrelevant to other confounding factors (9, 13). The criteria for the selection of IVs were as follows: (1) the potential IVs were selected with the significance threshold of single nucleotide polymorphisms (SNPs) related to each gut microbial genus within the range of loci (P<5.0×10-6); (2) Linkage disequilibrium (LD) between SNPs was calculated, and only the SNPs with the lowest P-value among SNPs with R2 < 0.001 (Clumping window size=10,000 kb) were retained; (3) SNPs with a minor allele frequency (MAF) ≤ 0.01 were excluded.

### 2.3 Statistical analysis

Data analysis in this study was performed using R (version 4.2.3) with the TwoSample MR (0.5.6) package and MRPRESSO (1.0). To verify whether there was a causation between exposure of gut microbiota and outcome LP, MR analysis was conducted using five methods such as inverse-variance weighted (IVW) (14), weighted median method (15), MR-Egger regression analysis (16), simple mode (12), and weighted mode (17). In addition, Cochran’s IVW Q was utilized to quantify the heterogeneity of IVs, and the false discovery rate test (FDR-q test) was performed (18), which is a multiple testing correction method used to control the expected proportion of false discoveries among the significant findings. It helps mitigate the risk of finding spurious associations due to testing multiple hypotheses. In the meantime, we performed a “Leave-One-Out” analysis by removing one genetic variant at a time and re-running the MR analysis. MR-egger intercepts to detect polytropy, in addition to which we performed MRPRESSO global tests to detect outliers and polytropy. This helps assess the influence of individual variants on the overall results. To assess the causal relationship between the gut microbiota and LP, we also carried out a reverse MR analysis of bacterial genera (phyla) found to be causally related to LP in the forward MR analysis. We used the GEO database (GSE52130) to retrieve LP-related expression chips, and used R-language “limma” package(3.52.2) for differential genes analysis, ggplot2 was used to visualize the results.

## 3 Results

**Figure 1:**
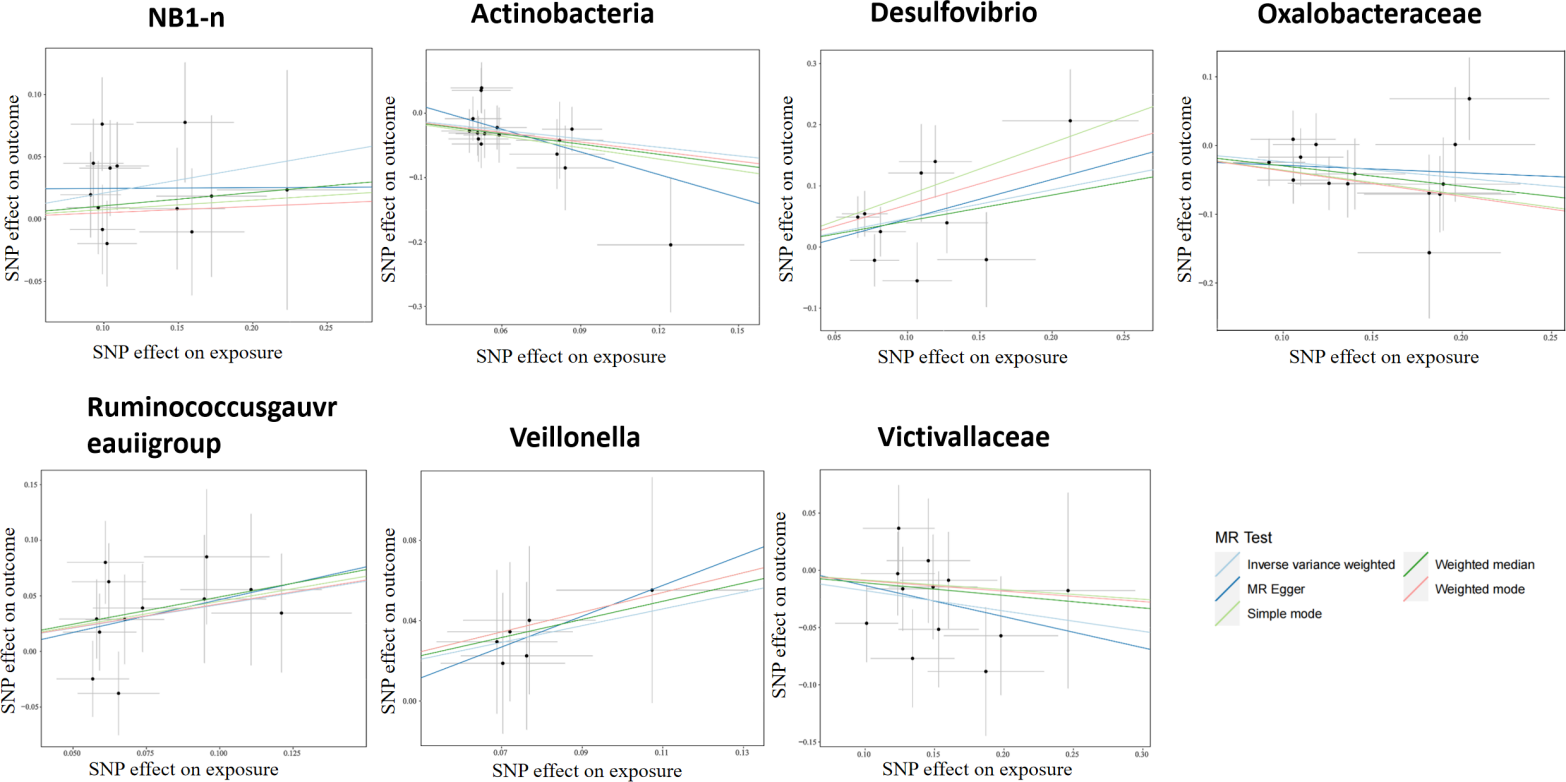
Scatter plot of two-sample MR results. The correlation between gut microbiota and LP was visualized in a scatter plot. In this plot, each black dot represented an SNP. With the correlation between SNP and exposure taken as the X axis, and the correlation between SNP and outcome taken as the Y axis, the slope of the drawn line marked the potential causal correlation of each method.

According to the selection criteria of IVs, 2763 SNPs were selected as IVs of 211 genera. As depicted in **Table 1**, 7 bacterial genera were identified by multiple MR methods, including *Oxalobacteraceae*, *Victivallaceae*, and *Actinobacteria*, which could restrain the onset of LP, showing a protective role, *Desulfovibrio*, *Veillonella, NB1n* and *Ruminococcus gauvreauii group* showed a promoting effect on lichen planus. IVW results showed that three bacterial genera were protective against LP: *Actinobacteria* (OR=0.64, 95%CI: 0.46-0.89, P=0.0093), *Oxalobacteraceae* (OR=0.78, 95%CI: 0.65-0.94, P=0.012), *Victivallaceae* (OR=0.83, 95% CI: 0.70-0.99, P=0.044), and the other four exhibited promoting effect on LP: *Desulfovibrio* (OR=1.59, 95%CI: 1.12-2.25, P=0.0082), *Victivallaceae* (OR=1.51. 95%CI: 1.02-2.25, P=0.039), *Ruminococcus gauvreauii group* (OR=1.52, 95%CI: 1.10-2.10, P=0.011), *NB1n* (OR=1.23, 95%CI: 1.02-1.49, P=0.034). Scatter plot showing the associations of the SNP effects on each bacterial genera against the SNP effects on LP (**Figure 1**).

**Table 1:** MR estimates for the association between gut microbiota and LP.

Bacterial genera	MR methods	No. of SNPs	OR	95%CI	P-value	q-value
*Oxalobacteraceae*	IVW	14	0.79	0.66-0.95	0.012	0.42
	Weighted median	14	0.74	0.58-0.95	0.018	0.63
	MR Egger	14	0.90	0.44-1.83	0.77	0.99
	Simple mode	14	0.70	0.46-1.05	0.11	0.97
	Weighted mode	14	0.69	0.46-1.04	0.1	0.97
*Victivallaceae*	IVW	12	0.84	0.70-1.00	0.044	0.51
	Weighted median	12	0.90	0.71-1.13	0.36	0.89
	MR Egger	12	0.76	0.33-1.74	0.53	0.99
	Simple mode	12	0.92	0.63-1.34	0.67	0.97
	Weighted mode	12	0.91	0.64-1.30	0.63	0.97
*Ruminococcus gauvreauii group*	IVW	12	1.53	1.10-2.11	0.011	0.72
	Weighted median	12	1.63	1.04-2.58	0.035	0.94
	MR Egger	12	1.80	0.47-6.90	0.41	1.00
	Simple mode	12	1.57	0.77-3.21	0.24	0.96
	Weighted mode	12	1.53	0.80-2.96	0.23	0.96
*Desulfovibrio(genus)*	IVW	10	1.6	1.13-2.26	0.0082	0.72
	Weighted median	10	1.53	0.96-2.44	0.07	0.94
	MR Egger	10	1.91	0.65-5.58	0.27	1.00
	Simple mode	10	2.34	1.06-5.15	0.06	0.96
	Weighted mode	10	1.99	1.02-3.90	0.07	0.96
*Veillonella*	IVW	6	1.52	1.02-2.26	0.039	0.81
	Weighted median	6	1.57	0.99-2.49	0.05	0.94
	MR Egger	6	2.15	0.09-49.65	0.66	1.00
	Simple mode	6	1.63	0.89-3.00	0.17	0.96
	Weighted mode	6	1.63	0.86-3.11	0.19	0.96
*Actinobacteria(phylum)*	IVW	15	0.64	0.46-0.90	0.0093	0.08
	Weighted median	15	0.59	0.37-0.92	0.021	0.18
	MR Egger	15	0.31	0.07-1.26	0.13	0.42
	Simple mode	15	0.55	0.26-1.15	0.13	0.90
	Weighted mode	15	0.61	0.34-1.10	0.12	0.90
*NB1n*	IVW	13	1.23	1.02-1.49	0.034	0.40
	Weighted median	13	1.11	0.85-1.46	0.44	0.94
	MR Egger	13	1.01	0.44-2.30	0.99	0.99
	Simple mode	13	1.08	0.70-1.66	0.74	0.89
	Weighted mode	13	1.05	0.68-1.63	0.83	0.87

MR, Mendelian randomization; LP, lichen planus; SNP, single nucleotide polymorphism; OR, odds ratio; CI, confidence interval; IVW, inverse variance weighted.

After the FDR-q test, *Actinobacteria* (OR=0.64, 95% CI: 0.46-0.89, P=0.0093, q=0.083) still displayed a significant protective role, while the other 6 gut microbiotas were suggested to correlate with LP in the IVW test, however, their correlation was insignificant after FDR-q test: *Oxalobacteraceae* (OR=0.78, 95%CI: 0.65-0.94, P=0.012, q=0.42), *Victivallaceae* (OR=0.83, 95%CI: 0.70-0.99, P=0.044, q=0.51), *Desulfovibrio* (OR=1.59, 95%CI: 1.12-2.25, P=0.0082, q=0.72), *Veillonella* (OR=1.51, 95%CI:1.02-2.25, P=0.039, q=0.81), and *Ruminococcus gauvreauii group* (OR=1.52, 95%CI: 1.10-2.10, P=0.011, q=0.72), *NB1n* (OR=1.23, 95%CI: 1.02-1.49, P=0.034, q=0.40). In addition, it was revealed that bacteria genera (phyla) in gut microbiota related to LP were screened by the forward MR analysis, but no significant correlation was found between these screened bacteria genera (phyla) and LP in the reverse MR analysis (LP and gut microbiota) (**Supplementary Table 1**).

**Figure 2:**
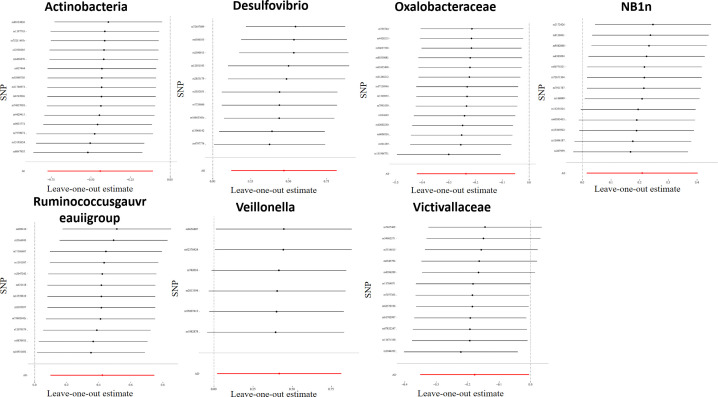
Leave-one-out plots for the causal association between gut microbiota and LP. After eliminating each SNP as an IV one by one, the overall trend did not change significantly, that was, no SNP with a great impact on the outcome among IVs.

For all instrumental variables, their F statistics individually range from 14 to 88, and all weak instrumental variables were excluded (**Supplementary Table 2**). Cochran’s IVW Q test exhibited no heterogeneity in these IVs (**Supplementary Table 3**). Furthermore, to identify potential heterogeneous SNPs, we performed a “Leave-one-out” analysis by omitting each SNP successively, and did not detect any single SNP that had a strong influence on the results (**Figure 2**). The q-value program was applied for false discovery rate (FDR) correction, and FDR-q<0.1 was deemed to be statistically significant after the pleiotropy test. However, even if FDR-q>0.1, a value of p<0.05 still suggested a certain correlation between gut microbiota and LP. Besides, based on the MR-Egger regression analysis results of scatter plot intercept, no significant horizontal pleiotropy was noted (P>0.05), and subsequent MR-PRESSO analysis did not find any significant outliers and no horizontal pleiotropy was found using the MR-PRESSO global test (P>0.05) (**Supplementary Table 3**). The funnel plot displayed a symmetrical and funneled shape, indicating little bias (**Supplementary Figure 2**).

It was observed that *Oxalobacteraceae*, *Victivallaceae*, and *Actinobacteria* could inhibit the occurrence of LP, exerting a protective effect, while *Desulfovibrio*, *Veillonella*, and *Ruminococcus dgauvreauii group* are causally related to the initiation of LP.

According to the screening conditions |fold change|>1.5 and P<0.05, a total of 340 genes were screened, including 260 upregulated genes, 90 downregulated genes. A volcano plot showed the distribution of differential genes (**Supplementary Figure 1**). The results revealed that the upregulated genes were mainly related to inflammation and the downregulated genes were mainly associated with various metabolic processes.

## 4 Discussion

This study conducted a two-sample MR analysis using the summary statistics of gut microbiota in the largest GWAS meta-analysis by the MiBioGen consortium and the summary statistics of LP data published by FinnGen on R8 to further evaluate the causal association between gut microbiota and LP. In this paper, three bacterial genera (*Actinobacteria*, *Oxalobacteraceae*, *Victivallaceae*) were revealed to exhibit protective effects, and four bacterial genera (*Desulfovibrio*, *Veillonella*, *Ruminococcus gauvreauii group, NB1n*) showed promoting impacts on LP. After the FDR-q test of the aforementioned six bacterial genera, *Actinobacteria* was found to be causally related to LP, while the correlation between the other five bacterial genera and LP was no longer significant.

There are a variety of microbial communities in the human intestine, which play a significant role in maintaining intestine-skin homeostasis. When the relationship between gut microbiota and the immune system is damaged, the skin may be affected, promoting the development of skin diseases. However, the mechanism of gut microbiota affecting skin health remains unclear (19, 20). Mahmud MR et al. systematically evaluated the microecology of healthy skin and intestine, the impact of probiotics on the gut microbiome, and their impact on skin health; The underlying mechanism of the intestine-skin axis and the link between the intestine and skin diseases such as atopic dermatitis were discussed. Dysbiosis of the gut microbiota leads to disruption of the intestinal barrier integrity, resulting in increased intestinal permeability; This allows microorganisms and toxins to enter the circulatory system and reach other target organs, including the skin (21). The consequence is a combination of systemic and local inflammation, thereby triggering skin disease. As an immune disease, LP provides conditions for the colonization of microorganisms in the oral cavity, especially after the occurrence of oral LP, while *Actinobacteria* may have a protective effect against LP *via* the intestine-skin axis. In this manuscript, the analysis through Mendelian randomization shown *Victivallaceae* may be associated with a decreased risk of incident LP. Analysis of the presence of SNPs in 12 presumptive competent-related genes showed rs7627405 associated with IL17, while from the results of differential genes expression analysis in LP, IL1F10, IL1B, IL1F5, IL1F9 are more correlated with occurrence of LP. The above evidence highlight that *Victivallaceae* may be involved in low incidences of LP through regulating the inflammatory response. But it still needs to be proved by further experiments (22, 23).

In this study, the causal relationship between gut microbiota and LP was determined using MR analysis to exclude the interference of confounding factors and the impact of reversed causality on causal inference. The intensity of IVs in MR analysis was ensured by parameter settings. Horizontal pleiotropy was detected and excluded using MR-PRESSO and MR-Egger regression intercept analysis. In addition, the application of the FDR-q test reduced the incidence of false positives. Meanwhile, there were also some shortcomings. First of all, this study employed summary statistics rather than the original data in the process of analysis. Hence, it was impossible to analyze the subgroups, and the lowest taxonomic level of the exposure data was genus. The current study could only reflect the protective impacts of *Actinobacteria* on LP. However, the MR analysis of *Bifidobacterium*, the most common probiotic in *Actinobacteria*, did not suggest a relationship with LP, and the specific bacteria that inhibited the onset of LP could not be further explored.

## 5 Conclusions

Altogether, the two-sample MR study revealed a certain causal association between gut microbiota and LP, and our MR analysis suggested that Actinobacteria in the gut microbiota had a protective effect on the initiation and development of LP (IVW: P<0.05, FDR-q<0.1). According to the intestine-skin axis theory, the dysbiosis of gut microbiota will affect the integrity of the intestinal barrier, leading to changes in intestinal permeability, so that microorganisms in the intestine can reach organs of the whole body including the skin through the circulatory system. Bacteria such as Actinobacteria (the dominant bacteria in the human intestine), Bacteroidetes, Firmicutes, and Proteobacteria account for about 90% of intestinal bacteria (24); In the meantime, Bifidobacterium and Actinobacteria can protect the intestinal barrier (25) Although our MR analysis did not suggest a correlation of Bifidobacterium in the intestine with LP (IVW: P>0.05), a randomized controlled trial (RCT) can be applied to further clarify the inhibitory effect of Actinobacteria on the onset of LP and its specific protective mechanism and further explore whether there is a potential mechanism related to the intestine-skin axis.

## Data availability statement

The original contributions presented in the study are included in the article/**Supplementary Material**. Further inquiries can be directed to the corresponding authors.

## Author contributions

MY, Y-LO: conceived the study and drafted the manuscript. L-YX, MA: data evaluation, manuscript preparation. MG, RF, RS: analyzed the data and revised the manuscript. L-LF, H-CF: conceived the study, designed the data evaluation, and prepared the manuscript. All authors contributed to the article and approved the submitted version.
