# Supplementary figures and images for: Correlations between gut microbiota and lichen planus: a two-sample Mendelian randomization study
Source: Front Immunol. 2023 Sep 12;14:1235982. doi: 10.3389/fimmu.2023.1235982 (PMC10521728; doi:10.3389/fimmu.2023.1235982)

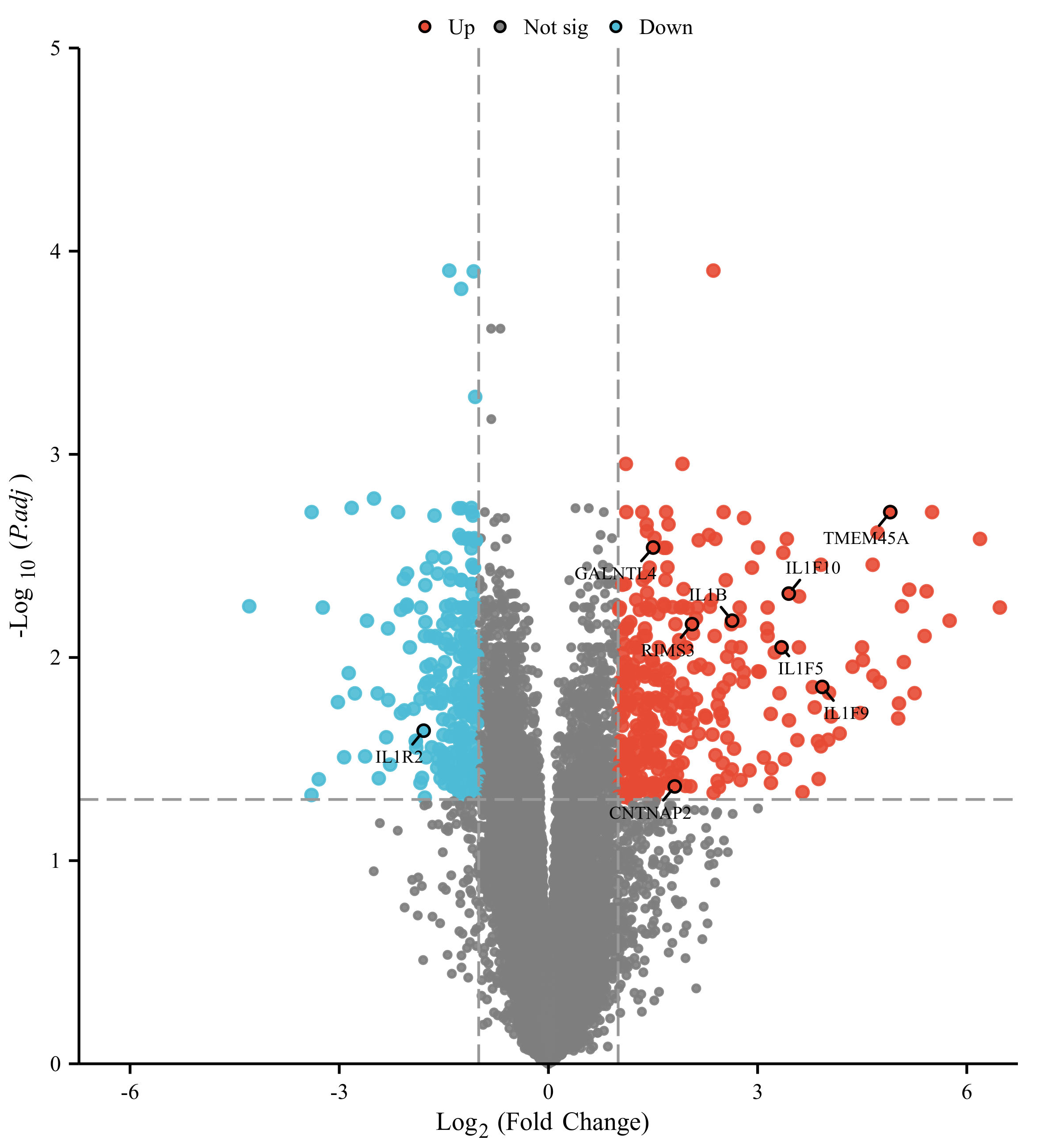

Supplement: Supplementary file 1 [file Image_1.tiff]

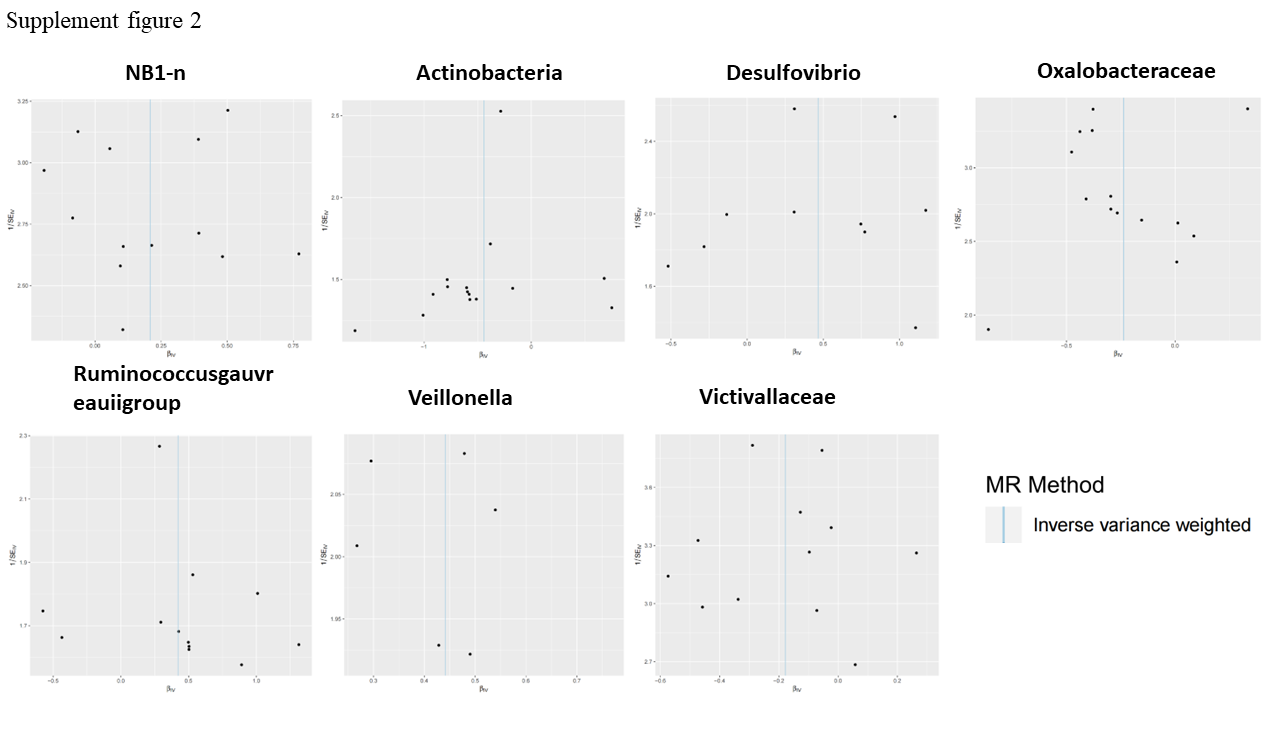

Supplement: Supplementary file 2 [file Image_2.tif]
